# Isolation, Antimicrobial Susceptibility Pattern and Associated Risk Factors of *Salmonella* Spp. in Slaughterhouses and Butchery Shops in Horo Guduru Wollega Zone, Ethiopia

**DOI:** 10.1002/vms3.71316

**Published:** 2026-11-07

**Authors:** Mitiku Wamile, Nagaro Damana, Lama Yimer, Megersa Gemechu, Abdu Muhammed

**Affiliations:** ^1^ Department of Animal Science Wollega University Shambu Ethiopia; ^2^ Guduru Research Center Wollega University Guduru Ethiopia; ^3^ School of Veterinary Medicine Wollega University Nekemte Ethiopia

**Keywords:** antimicrobial susceptibility pattern, antimicrobial resistance, Ethiopia, Horo Guduru Wollega Zone, *Salmonella* spp, slaughterhouse

## Abstract

**Background:**

Non‐typhoidal *Salmonella* (NTS) causes an estimated 93.8 million infections and 155,000 deaths annually worldwide; antimicrobial resistance (AMR) in *Salmonella* increasingly complicates treatment where antimicrobial use in livestock is poorly regulated. Therefore, this study determined the prevalence, antimicrobial susceptibility patterns and associated factors of *Salmonella* Spp. in slaughterhouses and butchery shops of Horo Guduru Wollega Zone, Ethiopia.

**Methods:**

A cross‐sectional study was conducted from October 2024 to June 2025. A total of 390 samples across three districts were collected, comprising bovine carcass swabs (*n = *180), faecal samples (*n = *180), cutting board swabs (*n = *15) and knife swabs (*n = *15). *Salmonella* spp. was isolated by ISO 6579:2002 and confirmed biochemically. Antimicrobial susceptibility was tested by Kirby–Bauer disc diffusion method. Data were analysed using descriptive statistics, Chi‐square tests and a mixed‐effects logistic regression model with random intercepts at the animal level.

**Results:**

*Salmonella spp*. was isolated from 42 of 390 samples (10.8%; 95% CI: 7.9–14.2). Carcass swabs had the highest isolation rate (16.1%, 29/180), followed by faecal samples (5.6%, 10/180). Among butchery environmental samples, cutting boards yielded 13.3% (2/15) and knives 6.7% (1/15). Carcass swabs carried 3.22 times higher odds of *Salmonella* contamination than faecal samples (AOR = 3.22, 95% CI: 1.52–6.82, *p < *0.001). Resistance was highest to tetracycline (83.3%), ampicillin (78.6%) and amoxicillin–clavulanic acid (71.4%); while ciprofloxacin (85.7%) and gentamicin (78.6%) showed the highest susceptibility. Multidrug resistance was identified in 64.3% (27/42) of isolates.

**Conclusions:**

*Salmonella* contamination is substantial among cattle carcasses and butchery environments in the Horo Guduru Wollega zone, with alarming levels of multidrug resistance. Higher contamination in carcass swabs suggests slaughter and handling practices drive cross‐contamination, while contamination of cutting boards and knives indicates hygiene gaps in retail butcheries, underscoring the needs for improved hygiene protocols, antimicrobial stewardship and consumer awareness.

## Graphical abstract

*Salmonella* contamination (10.8%) in Ethiopian cattle carcasses and butchery environments showed 64.3% multidrug resistance, with carcass contamination exceeding faecal carriage, implicating slaughter practices and highlighting urgent hygiene and stewardship needs.

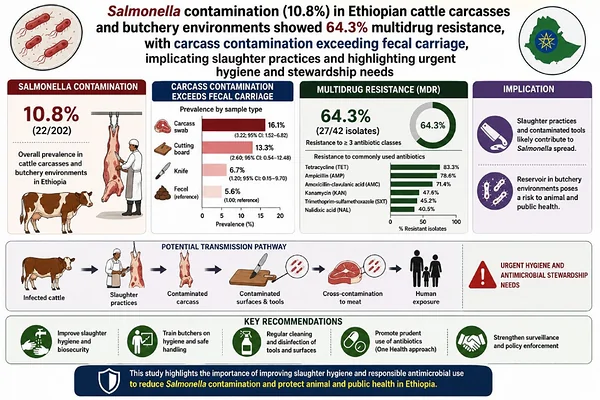

## 1 Introduction

Foodborne diseases remain a major public health challenge worldwide, imposing a substantial burden on human health, food security and economic development. According to the World Health Organization, unsafe food is responsible for an estimated 600 million cases of illness and 420,000 deaths annually worldwide (WHO 2015). Among the leading etiological agents, non‐typhoidal *Salmonella* (NTS) is increasingly recognised as a primary contributor to this global burden, accounting for approximately 93.8 million cases of human gastroenteritis and 155,000 deaths each year globally (Majowicz et al. 2010). In the United States alone, *Salmonella* causes an estimated 1.35 million infections, 26,500 hospitalisations and 420 deaths annually (Papp 2024). The genus *Salmonella *comprises two dominant species recovered from meat products: *Salmonella enterica* and *Salmonella bongori* with livestock reservoirs, including cattle, sheep and pigs, serving as primary sources of human infection (Ferrari et al. 2019; Bula‐Rudas et al. 2015).

Antimicrobial resistance (AMR) in *Salmonella* is a major concern in terms of human health, environmental sustainability and global food security.  *Salmonella*, the world's most prevalent foodborne pathogen, has gradually gained resistance to important drugs, complicating treatment efforts and leading to rising morbidity, mortality and economic costs. Multiple interrelated factors facilitate *Salmonella* transmission and AMR in Ethiopia, including antimicrobial misuse (overuse of antibiotics in human medicine and agriculture, coupled with inadequate regulatory compliance) and environmental contamination (agricultural runoff and treated sewage), slaughterhouse and processing practices (cross‐contamination via hides, intestinal contents, knives, cutting surfaces and worker hands), butchery hygiene gaps (contaminated cutting boards and knives), food handling and storage failures (improper temperature control and poor personal hygiene), traditional consumption practices (raw or undercooked beef dishes such as “kitfo”), and structural deficiencies (limited clean water, inadequate sanitation and weak enforcement of food safety regulations) (Kumar et al., 2025; Wabeto et al. 2017; Wales et al. 2011).

Humans acquire NTS infection through three principal transmission routes: direct contact with infected animals, environmental exposure and consumption of contaminated food (Heredia and García 2018). The consumption of undercooked or raw meat containing *Salmonella* provides a direct pathway through which this pathogen disseminates from food‐producing animals to human populations (Wabeto et al. 2017). Clinical manifestations of NTS infection range from asymptomatic carriage to severe systemic disease, including acute gastroenteritis, bacteraemia, meningitis and septic arthritis (Terentjeva et al. 2017). In immunocompromised individuals, particularly in resource‐limited settings, invasive NTS disease carries high case‐fatality rates, underscoring the clinical significance of this pathogen.

The global burden of NTS is disproportionately concentrated in low‐ and middle‐income countries (LMICs), where access to clean water, sanitary facilities and medical treatment is restricted (Kumar et al., 2025). The global burden of NTS is disproportionately concentrated in Africa. Reported infection rates reach 88% in Eastern Africa, 97% in Southern Africa and 87% in Western and Central Africa, compared to only 1% in Northern Africa (Ao et al. 2015). This geographic disparity reflects the convergence of multiple risk factors, including inadequate food safety regulations, weak enforcement mechanisms, limited public awareness and constrained healthcare infrastructure. Ethiopia carries a significant national burden of NTS, with human NTS prevalence estimates ranging from 6.2% to 13.6% (Eguale et al. 2015; Zewdu and Cornelius 2009). Concurrently, isolation rates of *Salmonella* from animal‐source foods across Ethiopian regions and commodities vary from 1.3% to 13.3% (Kore et al. 2017; Mekuriaw et al. 2016; Wabeto et al. 2017). Ethiopia carries a significant national burden of NTS, with human NTS prevalence estimates ranging from 6.2% to 13.6% (Eguale et al. 2015; Zewdu and Cornelius 2009). Concurrently, isolation rates of *Salmonella* from animal‐source foods across Ethiopian regions and commodities vary from 1.3% to 13.3% (Kore et al. 2017; Mekuriaw et al. 2016; Wabeto et al. 2017).

Multiple factors facilitate pathogen transmission in Ethiopian slaughterhouses and butcheries. A combination of inadequate food safety regulations, weak enforcement mechanisms, and limited public awareness creates an enabling environment for microbial contamination. According to the Ethiopian Ministry of Health, 60% of the national disease burden is attributable to suboptimal hygiene and sanitation practices (Ministry of Health 2005). The primary mechanisms of contamination include unsafe animal sourcing, cross‐contamination with raw materials, improper storage, poor personal hygiene among food handlers and insufficient temperature control (Linda and Irma, 2005). During slaughter, microorganisms contaminate bovine carcasses via transfer from external body surfaces and intestinal contents. Importantly, contamination sources extend beyond the animal itself to include fomites such as knives, cutting surfaces, cloths, ambient air, worker hands and processing equipment (Wales et al. 2011; Bhandare et al. 2007). Furthermore, food handlers in a carrier state or those experiencing acute *Salmonella* infection represent a significant vector for ongoing transmission within the food chain (Birhaneselassie and Williams 2013; Alemayehu et al. 2004; Ejeta et al. 2004). Traditional dietary practices in Ethiopia significantly amplify the risk of *Salmonella* infection. The consumption of raw or undercooked beef, particularly the traditional dish “*kitfo*” minced beef prepared either raw or lightly cooked, constitutes a direct route of pathogen transmission to humans (Andargie et al. 2008). This cultural practice dangerously intersects with the high prevalence of *Salmonella* in minced beef, thereby generating substantial community‐level transmission risk (Wabeto et al. 2017).

In Ethiopia, AMR in *Salmonella* is a growing concern. Studies from various regions have documented MDR prevalence ranging from 40.3% in Jimma (Takele et al. 2018) to 83% in Addis Ababa (Addis et al. 2011) and 100% in Wolaita Sodo (Wabeto et al. 2017). High resistance rates to commonly used antimicrobials, including tetracycline, ampicillin and trimethoprim–sulphamethoxazole, have been consistently reported across Ethiopian abattoirs and retail meat markets (Sibhat et al. 2011; Beyene et al. 2016). The widespread use of antimicrobial agents in food‐producing animals, coupled with limited regulatory oversight, has created favourable conditions for the emergence and dissemination of MDR *Salmonella* strains within the Ethiopian food chain. Recent Ethiopian studies confirm alarmingly high resistance rates among *Salmonella* isolates, with tetracycline resistance ranging from 73.3% to 100%, ampicillin resistance from 46.4% to 92.2%, and multidrug resistance (MDR) prevalence reaching 40.3% in Jimma (Takele et al. 2018), 50% in Asella (Beyene et al. 2016), 83% in Addis Ababa (Addis et al. 2011) and up to 100% in Wolaita Sodo (Wabeto et al. 2017). Similarly, high resistance to trimethoprim–sulphamethoxazole (47.6%–77.1%) and nalidixic acid (37.9%–56.9%) has been documented across multiple regions (Sibhat et al. 2011; Mekuriaw et al. 2016; Eguale et al. 2015).

Despite the significant economic burden imposed by *Salmonella* and the elevated public health risks associated with consuming contaminated meat products, epidemiological data from Horo Guduru Wollega Zone, Ethiopia, remain notably scarce. Therefore, this study determined the prevalence, antimicrobial susceptibility patterns and associated factors of *Salmonella* spp. in slaughterhouses and butchery shops of Horo Guduru Wollega Zone, Ethiopia. Given the zone's substantial cattle population and cultural practice of raw meat consumption, the absence of baseline data represents a critical gap for evidence‐based public health interventions.

## 2 Methodology

### 2.1 Study Area

The investigation was conducted from October 2024 to June 2025 in three districts of the Horo Guduru Wollega Zone, Oromia Region, Ethiopia: Guduru, Hababo Guduru and Abay Choman. Shambu, the capital of the study zone is located approximately 310 km west of Addis Ababa. The zone is characterised by a mixed agricultural system with substantial cattle production. The selected districts represent the primary livestock production areas within the zone.

### 2.2 Study Design and Sample Size

A cross‐sectional study design was employed to determine the prevalence and distribution of *Salmonella spp*. along with the slaughter‐to‐retail continuum. The minimum required sample size was calculated using the single population proportion formula for a cross‐sectional study:

$$n=\frac{Z^{2}\times P\times \left(1-P\right)}{d^{2}}\;$$

where;

- Z  = 1.96 (standard normal deviate for a 95% confidence level),
- P  = 0.5 (expected prevalence, assumed at 50% to maximise sample size and account for the absence of prior local prevalence data), and
- d  = 0.05 (desired absolute precision).

This yielded a minimum requirement of 384 samples. The final sample size was increased to 390 by adding 6 samples (approximately 1.5% increment) to compensate for potential sample loss, spillage or laboratory processing errors during transportation and culturing, ensuring the effective sample size remained adequate. Samples were systematically and proportionally distributed across all three districts and sample categories to ensure representative sampling. Samples were distributed proportionally across districts (130 per district) and across sample categories (180 carcass swabs, 180 faecal swabs and 30 environmental swabs) based on the availability of slaughterhouses and accessible butchery shops. Regarding sampling method, a systematic random sampling technique was employed at slaughterhouses: every third slaughtered animal was selected for paired carcass and faecal sample collection until the quota per district was reached. Environmental samples (cutting boards and knives) were collected from all available butcheries in each district using convenience sampling, as the number of operational butcheries was limited and pre‐identified.

### 2.3 Sampling Techniques and Sample Collection

Samples were systematically and proportionally distributed across all three districts and sample categories to ensure representative sampling. Four sample types were collected for a total of 390 samples: bovine carcass swabs (*n = *180), for which a 10 × 10 cm area on the flank, thorax, and breast of each slaughtered carcass was swabbed using sterile cotton swabs pre‐moistened in buffered peptone water (BPW) following ISO‐17604:2003; faecal samples (*n = *180), collected directly from the rectum of the same slaughtered cattle to determine the prevalence of *Salmonella* in the gastrointestinal tract; and butchery environmental swabs (knives and cutting boards, *n = *30), with fifteen swabs each collected from cutting boards and knives in retail butcher shops to assess environmental contamination in handling areas. Samples were collected from one municipal slaughterhouse per district (total of 3 slaughterhouses), with 60 carcass swabs collected from each slaughterhouse. All samples were placed into sterile containers supplemented with BPW, labelled with unique identification numbers, collection date and sampling location, transported on ice to Veterinary Laboratory, and processed within 24 h of collection.

### 2.4 *Salmonella* Isolation and Identification

*Salmonella* isolation followed the ISO 6579:2002 (ISO 2002) standard protocol. Samples in BPW were pre‐enriched at 37°C for 24 ± 3 h. Subsequently, 0.1 mL of pre‐enriched culture was transferred to 10 mL of Rappaport–Vassiliadis Soy broth (RVS) and incubated at 41.5 ± 1°C for 24 ± 3 h. In parallel, 0.1 mL of the same pre‐enriched culture was transferred to 10 mL of Müller–Kauffmann tetrathionate–novobiocin (MKTTn) broth and incubated at 37 ± 1°C for 24 ± 3 h. A loopful from each enrichment broth was streaked onto xylose lysine deoxycholate (XLD) agar and incubated at 37°C for 24 h. Suspected *Salmonella* colonies (appearing red with a black centre) were subcultured onto nutrient agar and confirmed through biochemical testing: triple sugar iron (TSI) agar, urease test, indole production, lysine decarboxylase activity and Voges–Proskauer reaction.

### 2.5 Antimicrobial Susceptibility Testing

The Kirby–Bauer disc diffusion method was performed on Mueller–Hinton agar following Clinical and Laboratory Standards Institute (CLSI) guidelines (CLSI 2005). Ten antimicrobial agents were tested (all from Oxoid, England): ampicillin (AMP, 10 µg), gentamicin (GEN, 10 µg), ciprofloxacin (CIP, 5 µg), ceftriaxone (CRO, 30 µg), trimethoprim–sulphamethoxazole (SXT, 25 µg), tetracycline (TET, 30 µg), kanamycin (KAN, 30 µg), nalidixic acid (NAL, 30 µg), amoxicillin–clavulanic acid (AMC, 30 µg) and cefotaxime (CTX, 30 µg). Following 16–18 h incubation at 37°C, inhibition zone diameters were measured in millimetres and interpreted as susceptible, intermediate or resistant according to CLSI interpretative standards. Quality control for antimicrobial susceptibility testing was performed using reference strains *Escherichia coli* ATCC 25922 and *Salmonella typhimurium* ATCC 14028 in accordance with CLSI recommendations. Interpretation of inhibition zone diameters followed updated CLSI interpretive criteria. Intermediate susceptibility results were not classified as resistant during MDR determination.

### 2.6 Data Analysis

Data were entered into Microsoft Excel spreadsheets and subsequently exported to R software (version 4.3, R Core Team, 2024) for statistical analysis. Descriptive statistics, including frequencies and proportions with 95% confidence intervals (CI), were calculated for all variables. Chi‐square tests assessed associations between sample type and *Salmonella* isolation status. To account for paired samples (carcass swab and faecal sample) collected from the same animal, variables with *p < *0.25 in univariate analysis were reanalysed using a mixed‐effects logistic regression model with random intercepts at the animal level, or alternatively, generalised estimating equations (GEE) with an exchangeable correlation structure. Statistical significance was set at *p < *0.05. Univariate logistic regression analysis calculated odds ratios (OR) and 95% CI to quantify the association between sample type and *Salmonella* positivity. Due to the small number of positive environmental samples (*n = *30 total), multivariable modelling and clustering adjustments (by slaughterhouse) were not feasible; this limitation is acknowledged. Statistical significance was defined as *p < *0.05.

## 3 Results

### 3.1 Overall *Salmonella* Isolation Rate

A total of 390 samples were collected from three districts of Horo Guduru Wollega Zone: Abay Choman (*n = *130), Guduru (*n = *130) and Hababo Guduru (*n = *130). The samples comprised four categories: bovine carcass swabs (*n = *180), faecal samples (*n = *180), cutting board swabs (*n = *15) and knife swabs (*n = *15). All samples were collected from October 2024 to June 2025 and processed within 24 h of collection. *Salmonella* was detected in 42 of 390 samples (10.8%; 95% CI: 7.9–14.2). Isolation rates differed substantially by sample category. Carcass swabs yielded the highest prevalence at 16.1% (29/180), followed by cutting board swabs at 13.3% (2/15), knife swabs at 6.7% (1/15) and faecal samples at the lowest rate of 5.6% (10/180). When analysed by district, each with 130 samples tested, the prevalence was 12.3% (16/130) in Abay Choman, 10.8% (14/130) in Hababo Guduru and 9.2% (12/130) in Guduru, with no statistically significant difference observed between districts (*p = *0.66) (Table 1).

**TABLE 1:** *Salmonella* isolation rates by district and sample type.

Variable	No. tested	No. positive (%)	95% CI	*p* value
**District**				
Abay Choman	130	16(12.3)	7.68–19.17	0.6
Guduru	130	12(9.2)	5.36–15.51	
Hababo Guduru	130	14(10.8)	6.52–17.35	
**Sample type**				
Carcass swab	180	29 (16.1)	11.2–22.1	
Faecal sample	180	10 (5.6)	2.9–9.8	< 0.001*
**Environment sample**				
Cutting board	15	2 (13.3)	2.3–38.2	
Knife	15	1 (6.7)	0.3–29.8	0.9
Total	390	42(10.8)	7.9–14.2	

* Significant

### 3.2 Mixed‐Effects Logistic Regression Analysis

Mixed‐effects logistic regression analysis (Table 2), accounting for paired carcass and faecal samples collected from the same animal through random intercepts at the animal level, demonstrated that carcass swabs carried significantly elevated odds of *Salmonella* contamination relative to faecal samples (AOR = 3.22, 95% CI: 1.52–6.82, *p = *0.002). McNemar's test for paired carcass and faecal samples (*n = *180 pairs) confirmed this finding: among 180 animals, 6 were positive in both sample types, 23 were positive only in carcass swabs, 4 were positive only in faecal samples and 147 were negative in both (*p < *0.001). Cutting boards also exhibited elevated odds, though not reaching statistical significance (AOR = 2.60, 95% CI: 0.54–12.48, *p = *0.234). Knife swabs showed odds comparable to faecal samples (AOR = 1.20, 95% CI: 0.15–9.70, *p = *0.864). District‐level differences were not significant (*p = *0.6). All estimates for environmental samples should be interpreted cautiously given the small number of positive samples.

**TABLE 2:** *Salmonella* isolation rates and mixed‐effects logistic regression analysis by district and sample type.

Variable	No. tested	No. positive (%)	95% CI	Crude OR (95% CI)	Adjusted OR (95% CI)	*p* value
**District**						
Abay Choman	130	16 (12.3)	7.68–19.17	1.00 (Ref)	1.00 (Ref)	0.6
Guduru	130	12 (9.2)	5.36–15.51	0.72 (0.33–1.59)	0.71 (0.32–1.58)	
Hababo Guduru	130	14 (10.8)	6.52–17.35	0.86 (0.40–1.85)	0.85 (0.39–1.83)	
**Sample type**						
Carcass swab	180	29 (16.1)	11.2–22.1	3.26 (1.54–6.90)	3.41 (1.60–7.28)	< 0.001*
Faecal sample	180	10 (5.6)	2.9–9.8	1.00 (Ref)	1.00 (Ref)	
**Environmental sample**						
Cutting board	15	2 (13.3)	2.3–38.2	2.15 (0.18–25.9)	2.10 (0.17–25.5)	0.9
Knife	15	1 (6.7)	0.3–29.8	1.00 (Ref)	1.00 (Ref)	
Total	390	42 (10.8)	7.9–14.2			

* Significant

### 3.3 Antimicrobial Susceptibility Profile

Antimicrobial susceptibility results for the 42 *Salmonella* isolates are presented in Table 3. Resistance to tetracycline (83.3%) was the most prevalent, followed by ampicillin (78.6%) and amoxicillin clavulanic acid (71.4%). In contrast, ciprofloxacin (85.7%), ceftriaxone (81.0%) and gentamicin (78.6%) demonstrated the highest susceptibility rates (Table 3). Intermediate susceptibility was notably elevated for nalidixic acid (21.4%), suggesting emerging reduced susceptibility to this agent.

**TABLE 3:** Antimicrobial susceptibility profile of 42 *Salmonella* isolates.

Antimicrobial agent	Abbr.	Conc.	Resistant *n* (%)	Intermediate *n* (%)	Susceptible *n* (%)
Tetracycline	TET	30 µg	35 (83.3)	2 (4.8)	5 (11.9)
Ampicillin	AMP	10 µg	33 (78.6)	1 (2.4)	8 (19.0)
Amoxicillin–clavulanic acid	AMC	30 µg	30 (71.4)	3 (7.1)	9 (21.4)
Kanamycin	KAN	30 µg	20 (47.6)	6 (14.3)	16 (38.1)
Trimethoprim–sulphamethoxazole	SXT	25 µg	19 (45.2)	4 (9.5)	19 (45.2)
Nalidixic acid	NAL	30 µg	17 (40.5)	9 (21.4)	16 (38.1)
Gentamicin	GEN	10 µg	5 (11.9)	4 (9.5)	33 (78.6)
Ceftriaxone	CRO	30 µg	5 (11.9)	3 (7.1)	34 (81.0)
Cefotaxime	CTX	30 µg	4 (9.5)	4 (9.5)	34 (81.0)
Ciprofloxacin	CIP	5 µg	3 (7.1)	3 (7.1)	36 (85.7)

### 3.4 Multidrug Resistance Prevalence

MDR, defined as resistance to three or more antimicrobial classes (Magiorakos et al. 2012), was identified in 27 of 42 isolates (64.3%) as shown in Figure 1. The predominant resistance pattern involved concurrent resistance to tetracycline, ampicillin and amoxicillin–clavulanic acid, frequently accompanied by resistance to additional agents. For MDR analysis, antimicrobial agents were grouped into the following classes: tetracyclines (TET), penicillins (AMP), β lactamase inhibitor combinations (AMC), aminoglycosides (GEN, KAN), fluoroquinolones (CIP), quinolones (NAL), folate pathway inhibitors (SXT) and cephalosporins (CRO, CTX). Intermediate susceptibility was not classified as resistant in MDR determination.

**FIGURE 1:**
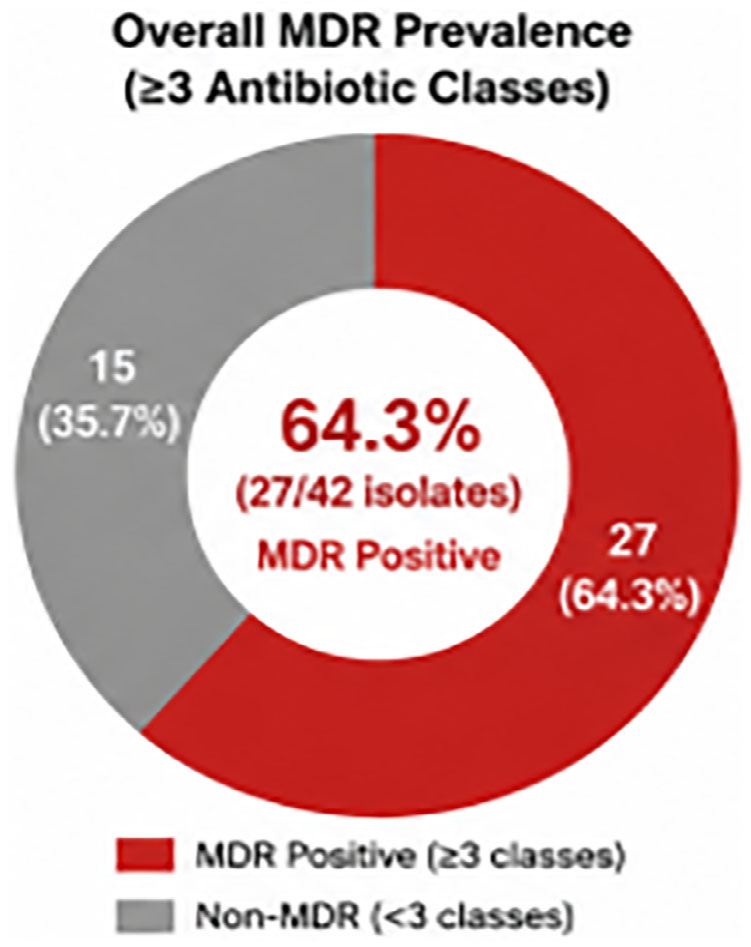
MDR prevalence.

The antimicrobial susceptibility testing revealed high resistance to tetracycline (83.3%), ampicillin (78.6%) and amoxicillin–clavulanic acid (71.4%). In contrast, low resistance was observed to ciprofloxacin (7.1%), as well as to ceftriaxone, cefotaxime and gentamicin (each approximately 10%). Consequently, high susceptibility was recorded for ciprofloxacin, ceftriaxone, cefotaxime and gentamicin (all exceeding 78%). The observed MDR prevalence indicates substantial circulation of resistant *Salmonella* isolates in slaughter and retail environments, representing a potential challenge for both veterinary and public health sectors.

## 4 Discussion

The overall 10.8% *Salmonella* isolation rate aligns closely with findings from comparable Ethiopian studies. Wabeto et al. (2017) reported a 12.5% prevalence at the Wolaita Sodo municipal abattoir, whilst investigations in Wolayta Sodo (Mekuriaw et al. 2016) and Bahir Dar (Muluneh and Kibret 2015) documented prevalences of 8.0% and 7.6%, respectively. The current findings are also comparable to reports from Jimma (11.3%), Takele et al. 2018) and Mekelle (7.3%), Teklebran et al. 2014).

Recent Ethiopian studies further support our findings. Geresu and Desta (2021) reported an 11.4% isolation rate from abattoir samples in Jimma, which is remarkably consistent with our overall prevalence. Similarly, a study conducted at the Dessie municipality abattoir by Tadesse et al. (2024) found an 8% prevalence of *Salmonella* species from cattle carcasses, with 8.3% from abattoir personnel. A recent study from Gondar Elfora Abattoir Tafere et al. (2025) reported a 12% prevalence in cattle samples, with carcass swabs showing 17.3% positivity. A systematic review and meta‐analysis (2026) estimated the pooled prevalence of *Salmonella* in Ethiopia at 6.7% (95% CI: 5.3%–8.1%), with substantial heterogeneity across regions.

However, it is substantially lower than reports from Gondar (35.6%) and another Ethiopian beef study (22.7%, Sibhat et al. 2011). Wabeto et al. (2017) attributed such inter‐study variability to differences in sampling methodologies, seasonal timing, microbiological techniques, slaughterhouse sanitation standards and mechanisms of cross‐contamination during carcass processing. These factors likely explain the observed range across Ethiopian abattoirs and underscore the need for standardised protocols to enable meaningful comparisons.

The significantly elevated contamination rate in carcass swabs (16.1%) compared to faecal samples (5.6%) suggests that cross‐contamination during slaughter may be an important transmission route. This finding is consistent with Tadesse et al. (2024), who reported that unwashed knives, carcasses and hands of butchers during slaughtering were significantly associated (*p < *0.05) with *Salmonella* found in carcasses at the Dessie municipality abattoir. Similarly, Geresu and Desta (2021) identified neatness of knives (OR = 7.6), source of contamination (OR = 8.44) and lack of job‐related training (OR = 5.50) as significant risk factors for carcass contamination in Jimma abattoirs. These findings gain support from Wabeto et al. (2017), who documented that microorganism transfer occurs from carcass exteriors, intestinal contents, knives, processing surfaces, worker hands and equipment during slaughter. Critically, our paired analysis provides a novel contribution to the literature: among matched animal pairs, carcass swabs were significantly more likely to be positive than corresponding faecal samples (McNemar's *p < *0.001). Although this finding is consistent with slaughter‐associated contamination, definitive causal attribution would require additional sampling of hides, water, worker hands and equipment sources not collected in this study.

The prevalence of tetracycline resistance (83.3%) and ampicillin resistance (78.6%) mirrors findings reported by Wabeto et al. (2017), who documented 83.9% and 46.4% resistance to these agents, respectively. The high‐ampicillin resistance observed in our study exceeds that reported by Wabeto et al. (2017), which may reflect differences in local antimicrobial usage patterns. Similarly, high resistance to both agents has been documented across Africa, including in Addis Ababa (Addis et al. 2011, Egypt (Sallam et al. 2014) and Jimma (Dabassa and Ketema 2012)). As highlighted in a recent review (Kumar et al., 2025), the overuse of antibiotics in human medicine and agriculture, coupled with inadequate regulatory compliance, exerts profound selection pressure favouring the emergence and persistence of resistant strains. These findings collectively underscore a continent‐wide challenge in livestock antimicrobial stewardship.

Recent studies confirm the continued high resistance to these agents. Seid et al. (2025) reported even higher resistance rates in northwest Ethiopia, with 100% of isolates resistant to tetracycline and 90.9% to ampicillin. Similarly, the Gondar Elfora Abattoir study Tafere et al. (2025) documented 58.82% resistance to cefoxitin, 41.2% to ampicillin and 35.3% to tetracycline. Geresu and Desta (2021) reported 58.3% resistance to tetracycline and 58.3% to ampicillin among abattoir isolates in Jimma, with 66.7% resistance to streptomycin. The resistance rate to amoxicillin–clavulanic acid (71.4%) is particularly alarming. Wabeto et al. (2017) reported lower resistance to this agent (42.9%) and cautioned that the recent introduction of amoxicillin–clavulanic acid combinations into African markets creates risk that resistance emergence may outpace clinical experience. Once amoxicillin–clavulanic acid‐resistant strains are established in human populations, therapeutic options will be substantially reduced, complicating management of invasive infections. This aligns with the One Health perspective, which emphasises that antimicrobial use in food animals directly impacts human clinical outcomes (Kumar et al., 2025).

Conversely, in vitro susceptibility to ciprofloxacin (85.7%) in the current study aligns closely with findings by Wabeto et al. (2017), who documented 87.5% susceptibility. Both studies suggest that this preserved efficacy stems from recent drug introduction and limited deployment in African countries. However, the emergence of any ciprofloxacin resistance (7.1%) and intermediate susceptibility to nalidixic acid (21.4%) represent early warning signals requiring careful monitoring. As noted by Kumar et al. (2025), fluoroquinolone‐resistant *Salmonella* strains are increasingly recognised globally, and their emergence in Ethiopia warrants prudent use of these agents to prevent further resistance amplification. Although ceftriaxone and cefotaxime demonstrated relatively high susceptibility rates, the presence of non‐susceptible isolates remains concerning because third‐generation cephalosporins are critically important antimicrobials for human medicine. The World Health Organization has classified cephalosporins as highest priority critically important antimicrobials (HP‐CIAs); therefore, any resistance to these agents constitutes a significant public health threat (Kumar et al., 2025). Further phenotypic and molecular investigations are needed to assess potential extended‐spectrum beta‐lactamase (ESBL) production.

The 64.3% MDR prevalence in the current study differs from the 100% MDR rate reported by Wabeto et al. (2017), though both represent alarmingly high levels. Kumar et al. (2025) recently emphasised that multidrug‐resistant *Salmonella* strains are now widespread across LMICs, driven by horizontal gene transfer (HGT) of mobile genetic elements (MGEs) carrying resistance determinants. The 64.3% MDR prevalence in the current study is consistent with recent Ethiopian findings. Tadesse et al. (2024) reported that 81.5% of *Salmonella* isolates from Dessie were resistant to three or more classes of antibiotics. Seid et al. (2025) found that 81.8% of isolates from northwest Ethiopia were multidrug‐resistant. Geresu and Desta (2021) reported that 58.3% of abattoir isolates and 66.7% of butcher shop isolates were resistant to two or more antibiotics. The Gondar Elfora study Tafere et al. (2025) also documented substantial MDR prevalence. These figures are alarmingly high and consistent with the 83% MDR prevalence reported in Addis Ababa (Addis et al. 2011). Our finding exceeds rates from Asella (50%, Beyene et al. 2016) and Jimma (40.3%, Takele et al. 2018), likely reflects regional differences in antimicrobial stewardship, regulatory enforcement and prescribing patterns. As Wabeto et al. (2017) emphasised, widespread MDR threatens economic viability and creates therapeutic impasses in human medicine. From a One Health standpoint, addressing this challenge requires integrated surveillance across human, animal and environmental sectors (Kumar et al., 2025).

The 10.8% prevalence in our study is consistent with the 11.4% reported by Geresu and Desta (2021) in Jimma but lower than the 17.3% carcass positivity reported by the Gondar Elfora study Tafere et al. (2025). These differences may reflect regional variations in hygiene practices, antimicrobial use patterns and slaughterhouse conditions. The systematic review and meta‐analysis (2026) identified substantial regional heterogeneity, with the highest pooled prevalence in Tigray (14.52%) and Oromia (8.14%), further supporting geographic variation in contamination rates.

### 4.1 Study Limitations

This investigation has several limitations warranting acknowledgement. First, *Salmonella* serotyping was not performed, limiting strain epidemiology characterisation, and hide swabs, worker hand swabs, water samples and air sampling were not collected, restricting definitive source attribution for cross‐contamination. Second, serotyping, minimum inhibitory concentration (MIC) testing, ESBL confirmatory assays and molecular characterisation of resistance determinants were beyond the present scope. Third, sampling was restricted to three districts, limiting generalisability beyond the study area. Fourth, the failure to collect environmental specimens (water, floor swabs and air) restricts our ability to fully assess the role of butchery environments in *Salmonella* transmission. Fifth, a single sampling season was employed, potentially missing seasonal variation in contamination rates.

## 5 Conclusions

This cross‐sectional investigation confirms a substantial prevalence of *Salmonella* contamination on bovine carcasses and within butchery environments of the Horo Guduru Wollega Zone, accompanied by alarming levels of MDR. Resistance to tetracycline, ampicillin and amoxicillin–clavulanic acid predominated, affecting most isolates. Carcass swabs demonstrated significantly higher contamination rates than paired faecal samples, suggesting that slaughter and handling practices contribute to processing contamination; isolation from cutting boards and knives documents critical hygiene gaps, though small sample sizes preclude firm conclusions. Importantly, while ciprofloxacin, ceftriaxone and gentamicin demonstrated relatively high in vitro susceptibility, the presence of any non‐susceptibility to third‐generation cephalosporins warrants continued surveillance and ESBL confirmatory testing.

Based on the above findings, the following recommendations are forwarded:

1. Veterinary professionals and livestock owners should promote responsible antimicrobial use based on susceptibility testing and avoid unnecessary prophylactic treatment.
2. Public health agencies should increase community awareness of safe meat handling, proper cooking and the risks associated with raw meat consumption.
3. One Health sectors should strengthen coordinated surveillance and AMR control through multisectoral collaboration.
4. Future studies should include larger environmental sampling, *Salmonella* serotyping, molecular resistance analysis and multi‐season surveillance to better understand transmission dynamics and resistance mechanisms.

## Author Contributions

**Mitiku Wamile**: conceptualisation, data curation, formal analysis, investigation, methodology, software, validation, visualisation, writing – original draft, writing – review and editing. **Nagaro damana**: data curation, investigation, validation, writing – review and editing. **Megersa Gemechu**: data curation, formal analysis, software, visualisation, writing – original draft, writing – review and editing. **Lema Yimer**: formal analysis, visualisation, writing – review and editing. **Abdu Muhammed**: data curation, investigation, writing – review and editing.

## Funding

This research was funded and received financial support from Wollega University (Ref.No. WU/184, 212/R.26), which covered costs associated with sample transportation, laboratory supplies, and technical assistance. The funding source had no involvement in the study design, collection, analysis, interpretation of data, writing of the report or the decision to submit the article for publication.

## Ethics Statement

Ethical approval was obtained from the Wollega University Ethical Review Board prior to sample collection. Informed verbal consent was secured from district authorities and butchery shop owners. All procedures complied with applicable institutional biosafety guidelines.

## Conflicts of Interest

The authors declare no conflicts of interests.

## Data Availability

The data supporting the findings of this study are available from the corresponding author upon reasonable request.
